# Individual Differences and Decision Making: When the Lure Effect of Gain Is a Matter of Size

**DOI:** 10.1371/journal.pone.0058946

**Published:** 2013-03-06

**Authors:** Barbara Penolazzi, Luigi Leone, Paolo Maria Russo

**Affiliations:** 1 Department of Psychology, University of Bologna, Bologna, Italy; 2 Department of General Psychology, University of Padova, Padova, Italy; 3 Department of Developmental and Social Psychology, “Sapienza” University of Roma, Roma, Italy; Inserm, France

## Abstract

The Iowa Gambling Task (IGT) is widely used in investigations of decision making. A growing number of studies have linked performance on this task to personality differences, with the aim of explaining the large degree of variability in healthy individuals' performance of the task. However, this line of research has yielded inconsistent results. In the present study, we tested whether increasing the conflict between short-term and long-term gains in the IGT can clarify personality-related modulations of decision making. We assessed performance on the original IGT as a function of the personality traits typically involved in risky decision making (i.e., impulsivity, sensation seeking, sensitivity to reward and punishment). The impact of these same personality traits was also evaluated on a modified version of the task in which the difference in immediate reward magnitude between disadvantageous and advantageous decks was increased, while keeping the net gain fixed. The results showed that only in this latter IGT variant were highly impulsive individuals and high sensation seekers lured into making disadvantageous choices. The opposite seems to be the case for participants who were highly sensitive to punishment, although further data are needed to corroborate this finding. The present preliminary results suggest that the IGT variant used in this study could be more effective than the original task at identifying personality effects in decision making. Implications for dispositional and situational effects on decision making are discussed.

## Introduction

A remarkable body of data on decision making has been collected using an apparently simple paradigm that requires the subject to select cards from decks that vary in both the probability and the extent of potential wins and losses – the Iowa Gambling Task (IGT, [1]). Crucially, this task entails resolving a conflict situation since, in order to achieve advantageous outcomes in the long-term, participants must select disadvantageous options in the short-term. The IGT was developed primarily to investigate impaired decision making in patients with frontal lobe damage; it has nonetheless also been used in countless studies that have investigated non-clinical samples. In this respect, a growing number of studies have recently linked IGT performance to personality differences as they have attempted to explain the large degree of variability in the performance of healthy individuals. Preliminary findings have suggested that specific personality traits can impact IGT performance, although results are often conflicting. Many studies have investigated this task by considering individual differences in the subjects' susceptibility to incoming rewards and punishments [2], coherent with IGT's reliance on reward/punishment schedules. Although some studies detected a negative impact of these personality traits on IGT performance, the findings are inconsistent [3]–[6]. Similarly incongruous results have been observed in studies that focus on the relationship between IGT performance and impulsivity – a trait that is typically involved when conflicting short-term and long-term incentives are embedded in the task. Despite findings in clinical samples that seem to consistently support a link between impulsivity and dysfunctional choice behavior, the data from non-clinical samples are far from conclusive. Some studies have shown impulsivity to have a significant detrimental effect on IGT performance [4], [7]–[11]; in other studies, however, no such relationship between impulsivity and IGT performance has emerged [5], [12], [13]. Inconsistent effects have also been reported for Zuckerman's Sensation Seeking Scale (SSS; [14]), which assesses how prone an individual is to exhibiting risky behaviors and making risky choices. Findings on this trait range from negative associations between IGT performance and specific facets of sensation seeking [8], [15], to null associations [3], [13], [16], or even to unexpected, positive associations between gains and the total SSS [17].

A full review of the research devoted to establishing associations between IGT performance and personality is beyond the scope of the present study. However, the brief literature survey presented above clearly suggests some measure of empirical inconsistency is present in the results thus far reported. Although the robustness of the IGT for investigating impaired decision making in clinical samples is well established, it could be argued that some specific features of the original IGT are less than ideal for detecting personality effects on healthy individuals' decision-making processes. In the present research, we hypothesize that a change in the original IGT reinforcement schedule aimed at increasing the degree of conflict between short-term and long-term gains could result in personality traits exerting more reliable effects on task performance. Therefore, we compared performance on the original IGT with performance on a modified version of the task introduced by Van den Bos and colleagues [18]. In this IGT variant (IGT-v), the difference in immediate reward magnitude between disadvantageous and advantageous decks was larger than in the original IGT. More specifically, the ratio of immediate rewards between disadvantageous and advantageous decks in the original IGT was 2∶1 (disadvantageous decks allowed one to gain €100 per selection, advantageous decks €50 per selection), whereas the ratio in the IGT-v was 6∶1 (disadvantageous decks allowed one to gain €300 per selection, advantageous decks €50 per selection). To assess the influence of the conflict between short-term and long-term gains on decision making, the immediate losses realized in disadvantageous decks were also increased to maintain a long-term net gain in those decks that would be identical to that of the original IGT.

Preliminary results, achieved in small, non-clinical samples, showed that this task variant severely impaired performance [18]. However, the effects of personality on this task have not yet been considered, despite the fact that the difficulties experienced when the conflict between short- and long-term gains increases effectively mimicked real-life situations in which personality has been shown to play a critical role in shaping decisions (e.g., health-risk circumstances, gambling contexts, etc.). To the best of our knowledge, the present study is the first to test the effects of sensitivity to rewards and punishments, impulsivity and sensation seeking on both IGT and IGT-v performance. We anticipate that the simple increase in immediate rewards/losses in disadvantageous decks in the IGT-v can disclose the effects of personality on decision making more effectively than can the reinforcement schedule of the original IGT.

Specifically, whereas it was difficult to predict sizeable effects of personality on IGT performance given the inconsistent evidence reported in the extant literature, we anticipated poorer performance among participants with high reward sensitivity in the IGT-v, since they can plausibly be described as being more sensitive to the increased immediate rewards of the disadvantageous decks. We expected similar poor performance on the IGT-v by highly impulsive participants, both because they can be more attracted to the increased immediate rewards of disadvantageous decks and because their distinctive planning deficits may be emphasized by the reward schedule of this task version. High sensation seekers were also expected to perform less efficiently on the IGT-v because the manipulation of the reinforcement schedule was thought to be likely to enhance the arousal produced by disadvantageous decks. In contrast, we anticipated better performance on the IGT-v among participants who were more sensitive to punishments, since the increased immediate losses of disadvantageous decks can induce such individuals to disregard such decks in order to avoid the anxiety generated by the prospect of losses.

## Methods

### Ethics statement

All participants gave their written, informed consent to take part in the study, which was performed after receiving approval from the University of Bologna, Department of Psychology Ethics Committee and in accordance with the Helsinki Declaration.

### Participants and Procedure

One hundred and seventy participants took part in the study. However, five participants were excluded due to problems related to questionnaire completion; therefore, the final sample included 165 volunteers (mean age: 26.47, sd ±7.14 years; 58.8% university undergraduate students, 41.2% workers). Participants were informed that they would be participating in a card game with fictitious payoffs and were randomly assigned to perform either the original (IGT) or the modified version (IGT-v) of the Iowa Gambling Task. They then completed a booklet that included several personality questionnaires (see below), which were pseudo-randomized across participants.

### Measures

#### Tasks

Decision making was assessed using two computerized versions of the Iowa Gambling Task – the IGT and the IGT-v.

In the IGT [1], participants were required to maximize their gains by choosing one card at a time from any of 4 decks, across 100 trials, starting from a specific amount of virtual money (€2,000). Participants were only aware that decks varied in their probability and magnitude of wins and losses, and that some decks, being less advantageous than others, would need to be avoided if they were to maximize their earnings. Two decks (i.e., decks C and D) were advantageous, leading the subject to immediately win and lose small amounts, with an eventual net gain (+ €250 per block of 10 cards); the remaining two decks (i.e., decks A and B) were disadvantageous, leading subjects to immediately win and lose large amounts, with an eventual net loss (− €250 per block of 10 cards). In accordance with the original IGT [1], the frequency of gains and losses in advantageous and disadvantageous decks was balanced. Namely, five of every ten trials generated a loss in decks A and C, whereas one in ten trials generated a loss in decks B and D. Participants did not know the rewards and punishments associated with each deck at the outset, but were able to learn it because they received visual feedback immediately after each selection, and were informed on the amount of money won or lost.

In the IGT-v [18] the ratio of immediate rewards between disadvantageous and advantageous decks differed from that in the original IGT. Specifically, in the original IGT the ratio was 2∶1, whereas in the IGT-v the ratio was 6∶1 (see *Introduction* section). In order to keep the net gain fixed with respect to that of the original IGT, the magnitude of immediate losses in the disadvantageous decks of the IGT-v was increased accordingly.

#### Personality questionnaires

The Behavioral Inhibition System (BIS) and Behavioral Approach System (BAS) Questionnaires [19], [20] were used to index sensitivity to punishments and rewards. These included 20 items, each rated on a 5-point scale and comprising four different subscales: (1) Sensitivity to Punishments (BIS-Anxiety), (2) positive responses to the occurrence/anticipation of rewards (BAS Reward Responsiveness), (3) persistence in the pursuit of reward (BAS Drive), (4) desire for novel rewards and a willingness to approach potentially rewarding situations (BAS Fun Seeking). Although a total BAS score, which sums the scores of the three BAS subscales, is often used, theoretical and psychometric evidence has suggested that it is best to consider these scales separately, as they refer to dissociable features of Reward Sensitivity [21], [22].

The Barratt Impulsiveness Scale (BIS-11, [23], [24]) was used to assess impulsivity. It consists of 30 items, each rated on a 4-point scale. The BIS-11 total score is computed by summing the scores of the three subscales that measure related facets of impulsivity: (1) a tendency to act without forethought in the spur of the moment (Motor Impulsiveness), (2) an orientation to the present or a lack of planning for the future (Non-Planning), and (3) difficulty in maintaining attention or concentration (Attentional Impulsiveness).

The Sensation Seeking Scale, version V (SSS-V, [14]), was used to measure individual preferences for arousing and stimulating activities and experiences. It consists of 40 pairs of antithetical items and assesses the subject's tendency to engage in both dangerous/adventurous activities and new mental and sensory experiences, the subject's interest in socially and sexually disinhibited activities, and the subject's aversion to routine and repetitive activities. Higher scores in the SSS-V indicate a higher degree of sensation seeking.

### Data analysis

In both task versions, in order to check for the presence of learning effects, analyses of trials grouped in five blocks (each block consisting of 20 consecutive trials) were performed using the *net score* as a dependent variable. This was computed as the difference between advantageous and disadvantageous deck selections, with a positive net score indicating more advantageous selections. We first examined participants' performance independent of personality variables through an Analysis of Covariance (ANCOVA), specifying the five *Blocks* of net score as a within-group factor, *Gender* and *Task Version* (IGT vs. IGT-v) as between-group factors, and *Age* as a covariate (being this significantly different between females and males, see *Results* section). We then considered performance as a function of personality by performing, for each of the six measured traits, an ANCOVA with the same factors as above and the additional factor *Group*, obtained by splitting the volunteers according to the median score of the trait. Type-1 error for the 6 ANCOVAs was controlled for by setting the significance threshold at the level of *p* = 0.008 (i.e. *p* = .05/6). The Greenhouse – Geisser correction was applied when necessary and significant effects were explored using Tukey HSD post-hoc tests for unequal samples. In addition, Pearson correlations between personality traits and total net score were computed for each version of the task. To gauge the unique contribution of each trait to behavioral decision making, hierarchical regression analyses were performed – separately for each task version – by regressing the total net score on the self-reported traits. Gender and age were entered into the equation first, as statistical controls.

In order to detect the presence of effects in the gain-loss frequency dimension (i.e., decks A and C: loss in 50% of the trials vs. decks B and D: loss in 10% of the trials), we analyzed the number of selections from each deck during the entire task performance. In this regard, particular attention was devoted to the preference, if any, for deck B – an effect known as “prominent deck B” [10], [25], an index of irrational decision making sometimes reported among healthy participants. We therefore first examined participants' performance independent of personality variables, through an ANCOVA, specifying the *4 Deck Selection* as a within-group factor, *Gender* and *Task Version* as between-group factors, and *Age* as a covariate. We then considered performance as a function of personality by performing ANCOVAs analogous to those previously described for the analyses in the gain-loss magnitude dimension.

## Results

Table 1 shows the descriptive statistics for the total sample and for males and females separately. T-tests revealed that females were slightly younger and had higher BIS-Anxiety scores than did males, whereas males had significantly higher SSS-V scores. No gender differences were detected in task performance for either IGT version. Participants assigned to the original IGT (*N* = 84) and to IGT-v (*N* = 81) were equivalent in terms of gender distribution, age, and personality traits (all *p*s >0.05).

**Table 1:** Descriptive statistics (mean ± SD) of personality measures and task performance.

Measures	Total Sample	Males	Females	t-value
**Age**	26.47±7.14	28.46±7.90	24.60±5.79	3.56**
**BIS-11**	60.90±8.99	60.94±8.69	60.87±9.31	0.05
**Motor Impulsiveness**	19.70±4.15	19.51±4.24	19.87±4.08	−0.55
**Non-Planning**	24.51±4.19	24.61±4.34	24.42±4.06	0.29
**Attentional Impulsiveness**	16.69±3.51	16.81±3.34	16.58±3.69	0.43
**BIS-Anxiety**	23.80±5.05	22.13±5.15	25.38±4.43	−4.33**
**BAS Reward Responsiveness**	20.36±2.91	20.37±2.87	20.34±2.96	0.74
**BAS Drive**	12.04±3.20	12.19±3.02	11.91±3.36	0.56
**BAS Fun Seeking**	11.21±3.40	10.36±3.23	11.07±3.57	0.55
**SSS-V**	18.24±5.99	19.21±5.92	17.31±5.94	2.05*
**IGT-Net score**	10.12±24.49	14.35±27.40	6.27±21.08	1.50
**IGT-v Net score**	−3.16±26.40	−6.45±27.64	0.05±25.06	−1.1

*Notes*. BIS-11: Barratt Impulsiveness Scale; BIS: Behavioural Inhibition System; BAS: Behavioural Approach Systems; SSS-V: Sensation Seeking Scale-v; IGT: Iowa Gambling Task; IGT-v: variant of the Iowa Gambling Task; Net score is computed as difference between advantageous and disadvantageous deck selections; Total Sample: N = 165, Males: N = 80, Females: N = 85; IGT: N = 84, Males = 40, Females: N = 44; IGT-v: N = 81, Males = 40, Females: N = 41; *p<.05, **p<.001.

### Net score analyses

**Figure 1:**
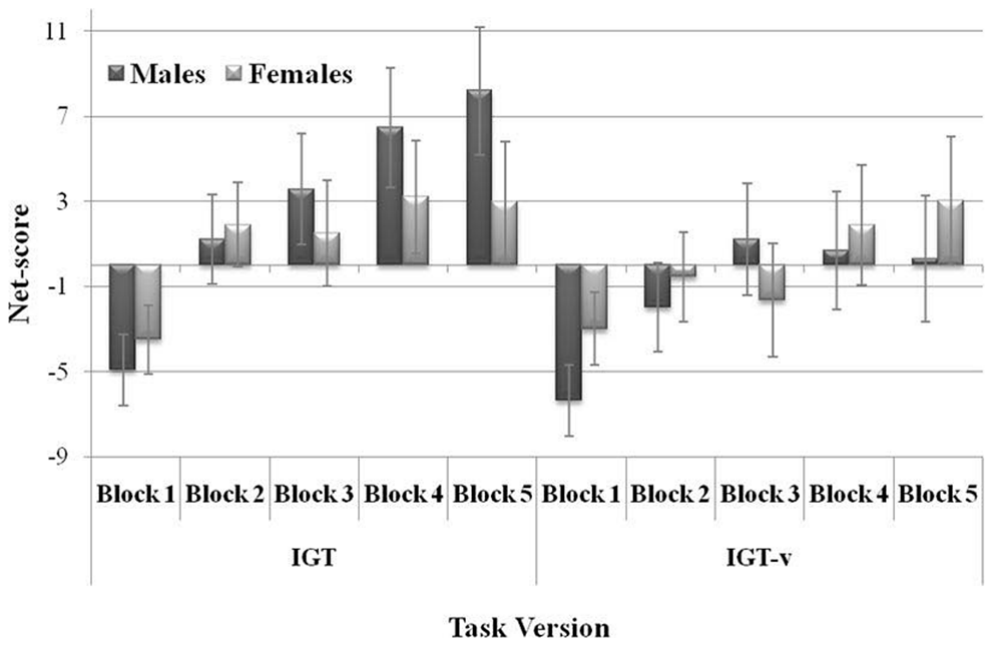
Net scores in the five blocks as a function of IGT version and Gender. Mean net scores in the five blocks of the original (IGT, on the left) and modified (IGT-v, on the right) task versions for males and females. Vertical bars denote +/−0.95 confidence intervals.

The ANCOVA focused on learning effects revealed Block to have a significant main effect (*F*_(4,640)_  = 5.15, *η^2^* = 0.03, *p* = 0.001: the first block showed a significantly lower mean net score compared with the net score of all other blocks: the second block showed a mean net score significantly lower compared with the net scores of the fourth and fifth blocks, ; and the third block showed a net score lower than that of the fifth block (m±se of the net score in the five blocks: −4.43±0.42; 0.14±0.52; 1.17±0.65; 3.06±0.69; 3.62±0.74; p<0.01, Tukey HSD post-hoc tests). The main effect of Task Version was also significant (*F*(_1,160)_  = 11.67, *η^2^* = 0.07, *p* = 0.001), with the IGT-v characterized by a significantly lower mean net score per block (i.e., total net score divided by five blocks) than that of the original IGT (m±se: −0.64±0.56 and 2.06±0.55, respectively). The two-way Block by Task Version interaction was not significant (*F*_(4,640)_  = 1.63, *η^2^* = 0.01, ns), a result that suggests that participants learned the advantageous choice strategy in both versions, considering the Block main effect. The two-way Gender x Task Version interaction approached statistical significance (*F*_(1,160)_  = 3.23, *η^2^* = 0.02, *p* = 0.07): females performed comparably in both versions (Females, IGT net score  = 1.25±0.71; Females, IGT-v net score  = 0.02±0.73; ns), whereas males performed significantly better in the classic IGT than in the IGT-v (Males, IGT net score  = 2.84±0.87; Males, IGT-v net score  = −1.26±0.87; *p*<0.01). In addition, a complex, three-way interaction, Gender x Task Version x Block, was found to be significant (*F*_(4,640)_  = 2.62, *η^2^* = 0.02, *p* = 0.041, see Figure 1). Post-hoc comparisons showed gender differences in the fifth block to be a function of IGT version: females' performance was the same irrespective of task version (females' IGT net score in the fifth block  = 2.97±1.44; females' IGT-v net score in the fifth block  = 3.02. ±1.51), whereas a remarkable decline in performance was observed for males in IGT-v as compared to IGT (males' IGT net score in the fifth block  = 8.19±1.51; males' IGT-v net score in the fifth block  = 0.31±1.53; *p*<0.001).

ANCOVAs that included the effects of personality variables did not yield significant results for the BIS-BAS scales (all *p*s >0.05).

**Figure 2:**
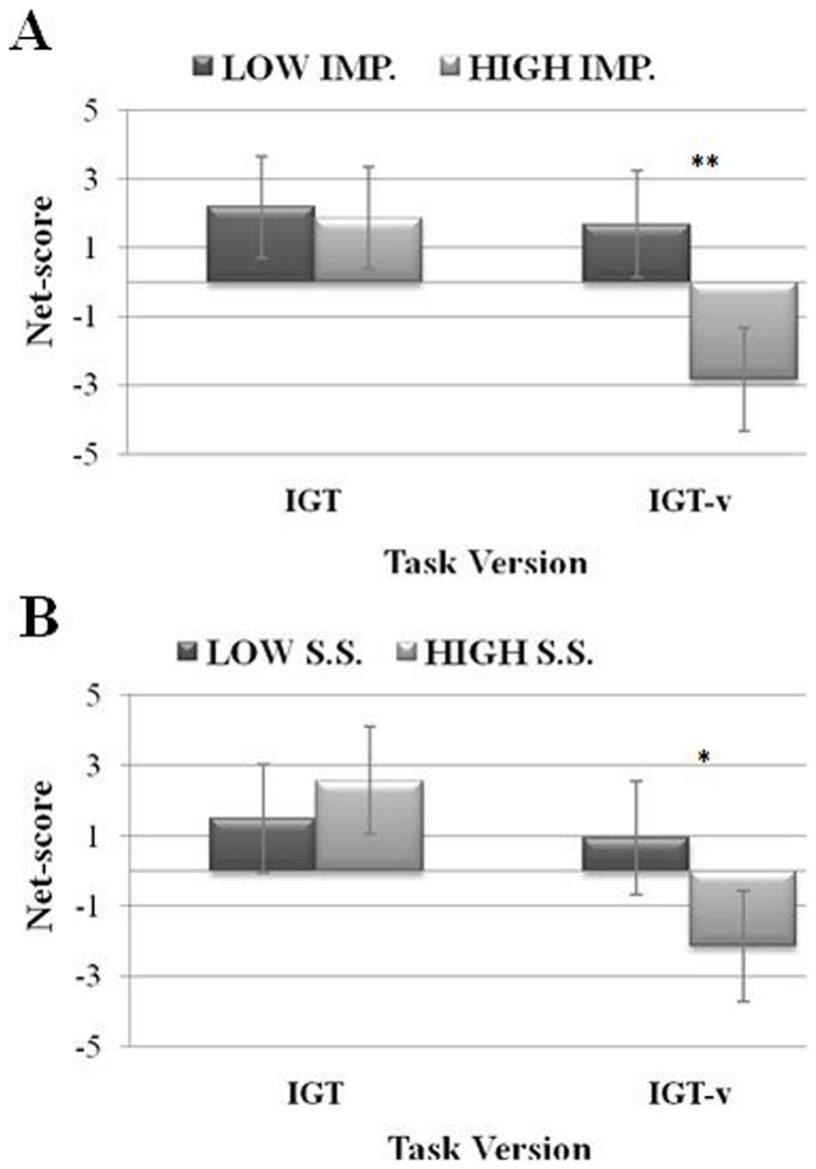
Net scores as a function of IGT version for Impulsivity and Sensation Seeking levels. Mean net scores in the original (IGT) and modified (IGT-v) task versions for participants with *Low vs. High Impulsivity* levels (A) and participants with *Low vs. High Sensation Seeking* levels (B). Vertical bars denote +/−0.95 confidence intervals, *p≤0.05, **p≤0.01.

We did detect, however, a main effect of Impulsivity (*F*_(1,156)_  = 9.89, *η^2^* = 0.06, *p* = 0.002), as measured by the BIS-11, which was qualified by the significant Impulsivity x Task Version interaction (*F*_(1,156)_  = 7.52, *η^2^* = 0.05, *p* = 0.007, see Figure 2A). Post-hoc tests showed that highly impulsive individuals were characterized by poorer performance than less impulsive individuals only in the IGT-v (*p* = 0.0003).

The Impulsivity x Block interaction was also significant (*F*_(4,624)_  = 4.96, *η^2^* = 0.03, *p* = 0.001), indicating that more impulsive participants recorded significantly lower net scores than did less impulsive participants only in the fourth (*p* = 0.009) and fifth blocks (*p* = 0.01). Further analyses were performed with the purpose of establishing the possible contribution of the three BIS-11 subscales to the effect found for total Impulsivity. Such analyses revealed that Attentional Impulsiveness did not show any significant effect (all *p*s >0.05). Motor Impulsiveness showed a significant interaction with Task Version (*F*_(1,156)_  = 4.20, *η^2^* = 0.03, *p* = 0.042), similar to that found for total Impulsivity. Non-Planning showed the same three significant effects identified for total Impulsivity, thus emerging as the principal source of total Impulsivity effects – i.e., Non-Planning main effect (*F*_(1,156)_  = 16.60, *η^2^* = 0.10, *p* = 0.0001); Non-Planning x Task Version (*F*_(1,156)_  = 4.01, *η^2^* = 0.02, *p* = 0.047); and Non-Planning x Block (*F*_(4,624)_  = 3.07, *η^2^* = 0.02, *p* = 0.02).

A noteworthy interaction between Sensation Seeking and Task Version was found (*F*_(1,156)_  = 6.95, *η^2^* = 0.04, *p* = 0.009; see Figure 2B). Although it failed to achieve full statistical significance after our correction (*p* = 0.008), this finding was explicitly anticipated in our hypotheses, and thus was considered to be worth mention. Post-hoc tests revealed that high sensation seekers performed worse than low sensation seekers only in the IGT-v (*p* = 0.02).

Correlations among personality measures and the total net score for the two IGT versions are reported in Table 2.

**Table 2:** Pearson correlation coefficients between self-report and behavioural variables.

	1	2	3	4	5	6	7	8	9
**1. BIS-11**	.78								
**2. Motor Impulsiveness**	.80**	.68							
**3. Non-Planning**	.80**	.48**	.59						
**4. Attentional Impulsiveness**	.66**	.29**	.29**	.67					
**5. BIS-anxiety**	.11	.04	−.02	.25**	.84				
**6. BAS Reward Responsiveness**	.05	.12	−.15*	.16*	.28**	.71			
**7. BAS Drive**	.14	.25**	.03	.03	−.04	.34**	.79		
**8. BAS Fun Seeking**	.54**	.55**	.33**	.33**	.01	.36**	.32**	.79	
**9. SSS-V**	.37**	.35**	.24**	.24**	−.21**	.13	.26**	.46**	.78
**IGT Net score**	−.08	−.05	−.23*	.10	−.14	.21	−.07	.05	.17
**IGT-v Net score**	−.44**	−.30**	−.39**	−.29**	.19	−.00	−.12	−.28*	−.36**

*Notes*. Cronbach's Alpha reliability indexes are reported in the main diagonal; BIS-11: Barratt Impulsiveness Scale; BAS: Behavioural Approach Systems scale; SSS-V: Sensation Seeking Scale-v; IGT: Iowa Gambling Task; IGT-v: variant of the Iowa Gambling Task; * p≤0.05, **p≤0.01; bold values indicate significant correlations between self-report measures and task performance.

Total net score in the original IGT was only negatively associated with the Non-planning facet of Impulsivity. In contrast, the total net score in the IGT-v showed significant negative correlations with several personality dimensions, including Sensation Seeking as well as Impulsivity total score and its three subscales, Motor Impulsivity, Non-Planning and Attentional Impulsivity. In line with these findings, we also found a negative correlation between IGT-v net score and BAS Fun Seeking.

Hierarchical multiple regression analyses, which aimed to test the net effects of the traits under investigation on behavioral decision making, further showed that IGT-v was more effective than IGT at revealing the influence of personality on decision making. For the original IGT, the overall regression model (corrected *R^2^* = 0.107; *F*_(10,73)_  = 1.99, *p* = 0.047) showed that participants scoring lower in the Non-Planning facet of Impulsivity performed better (*β* = −0.32, *t* = −2.30, *p* = 0.02). As regards the IGT-v, the traits included in the model accounted for more than twice the variance of performance on the IGT-v than the same traits accounted for in the model of performance on the IGT (corrected *R^2^* = 0.236; *F*_(10,70)_  = 3.47, *p* = 0.001). For the IGT-v, the Non-Planning (*β* = −0.28, *t* = −2.48, *p* = 0.01) and Attentional Impulsivity (*β* = −0.24, *t* = −2.03, *p* = .046) facets negatively impacted performance, whereas BIS-Anxiety positively impacted performance (*β* = 0.27, *t* = 2.20, *p* = 0.03).

### Deck selection analyses

**Figure 3:**
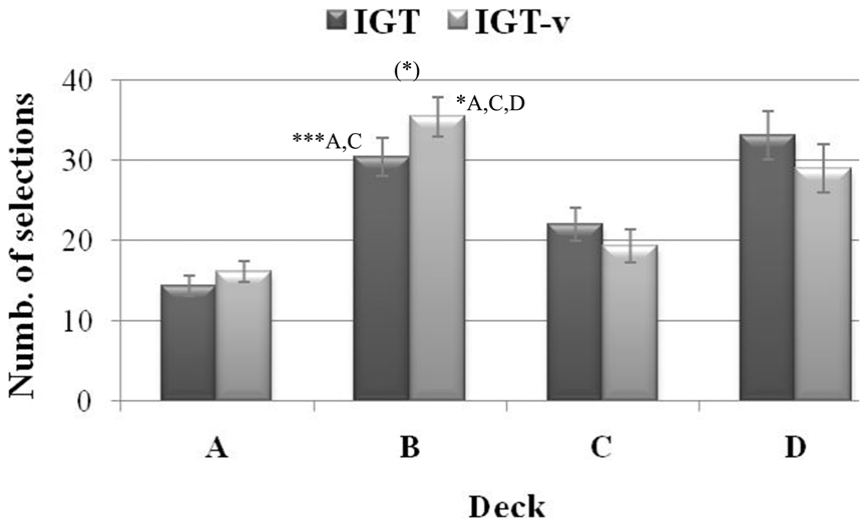
Selection from each deck as a function of IGT version. Mean number of cards chosen from each deck in the original (IGT) and modified (IGT-v) task versions. Vertical bars denote +/−0.95 confidence intervals, (*) *p* = 0.055,*p≤0.05, ***p≤0.001.

The ANCOVA that focused on deck selection throughout the task revealed a significant main effect of Deck Selection (*F*_(3,480)_  = 6.34, *η^2^* = 0.04, *p* = 0.002), which was more clearly qualified by the significant Deck Selection x Task Version interaction (*F*_(3,480)_  = 4.87, *η^2^* = 0.03, *p* = 0.007, see Figure 3).

Post-hoc tests revealed that, in the IGT, there were significantly more selections made from decks B and D than there were made from decks A and C (all *p*s <0.001). Participants generally preferred decks with high-frequency gains – B and D – compared with decks with low-frequency gains – A and C. Conversely, in the IGT-v, the “prominent deck B effect” [25] was present, as there were significantly more selections made from this deck as compared with all other decks, including deck D (all *p*s <0.05). In addition, by directly comparing the two task versions, a marginally significant effect (*p* = 0.055) was found for deck B in particular, with more selections from this deck in the IGT-v than in the IGT.

**Figure 4:**
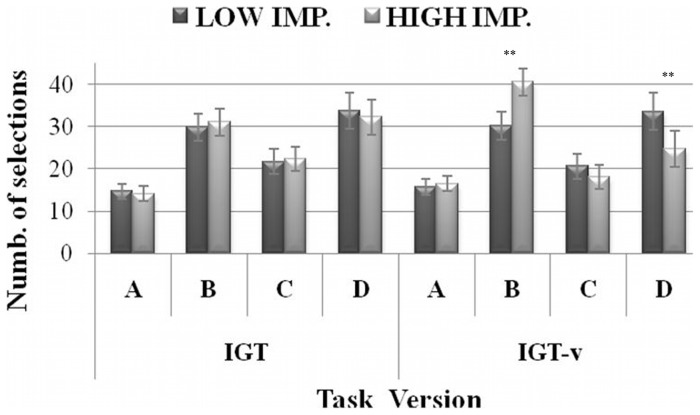
Selection from each deck as a function of IGT version for Impulsivity levels. Mean number of cards chosen from each deck, in the original (IGT, on the left) and modified (IGT-v, on the right) task versions for participants with *Low vs. High. Impulsivity* levels. Vertical bars denote +/−0.95 confidence intervals, **p≤0.01.

ANCOVAs that included the effects of personality variables showed significant results only for Impulsivity, which interacted with Deck Selection (*F*_(3,468)_  = 5.80, *η^2^* = 0.04, *p* = 0.003). Post-hoc tests revealed that highly impulsive participants made significantly more selections from deck B (*p* = 0.01) and significantly fewer selections from deck D (*p* = 0.04) compared with less impulsive participants. Although the Impulsivity x Task Version x Deck selection interaction can be considered only marginally significant (*F*_(3,468)_  = 3.56, *η^2^* = 0.02, *p* = 0.027, see Figure 4) after the *p*-level correction (significance threshold: *p* = 0.008), it did help in qualifying the Impulsivity x Deck Selection interaction.

Indeed, post-hoc tests in the three-way interaction showed that the differences between high and low impulsive individuals were present exclusively in the IGT-v (*p* = 0.002 for deck B; *p* = 0.01 for deck D). In addition, whereas low impulsive participants did not show differences in deck selection as a function of Task Version, highly impulsive individuals made significantly more selections from deck B (*p* = 0.006) and significantly fewer selections from deck D (*p* = 0.04) in the IGT-v as compared with the classic IGT.

## Discussion

The present study was devoted to exploring the impact of personality on decision making by using a variant of the IGT, the IGT-v [18], in which the immediate rewarded value of disadvantageous decks was increased while the long-term net gain of these decks was kept intact. This manipulation of the reinforcement schedule was aimed at magnifying the conflict between short- and long-term gains, and preliminary results showed it severely negatively impacted healthy participants' performance [18]. To the best of our knowledge, the IGT-v has not yet been used to investigate personality differences that might affect decision making in particular; therefore we have specifically addressed this issue in the present study.

The significantly worsened performance of participants on the IGT-v as compared with their performance on the original IGT supports the notion that, independent of personality traits, the more alluring pay-off of its disadvantageous decks allows it to impair long-term, efficient decision making, as previously reported by Van den Bos and colleagues [18]. Likewise, the fact that we identified the “prominent deck B” effect [25] exclusively in the IGT-v suggests that it allows for easier identification of suboptimal decision making tendencies than the original IGT.

Males and females showed different performance along the gain-loss magnitude dimension. Previous studies had reported gender-based differences in IGT performance, with males outperforming females in this task [13], [17], [26], [27]. Although such a pattern was not clear in the results of the present study, we did identify a different learning effect in task performance as a function of both task version and gender. Specifically, females performed comparably in the two task versions, whereas males performed significantly worse in the IGT-v than in the classic IGT (with much of the decline occurring in the fifth block), showing to be particularly sensitive to the increased conflict between short-term and long-term gains. Since the more frequently reported personality differences between genders involve the same traits that are considered likely to affect decision making in the present study (i.e., impulsivity, sensation seeking, anxiety), it is reasonable to link the gender differences in performing the task to personality differences. Although the present data are insufficient for drawing strong conclusions, they offer a hint for future studies into the relationship between gender and personality and how that relationship might affect decision-making behaviors.

Turning attention to the effects of personality differences, although further data are needed to corroborate the preliminary findings of the present study, our results suggest that the IGT-v could be a more sensitive tool than the classic IGT in disclosing the effects of specific traits on choice behavior. We reason that the classic IGT generates a conflict between short- and long-term gains that can be too weak to make individual differences in personality relevant to how choices are made. The only personality trait that appeared to negatively influence performance on the original IGT was Impulsivity – the Non-Planning facet, in particular. The fact that such a component affected performance on the IGT-v, as well, and that it did so to an even greater extent, highlights the fundamental role a lack of planning (or a present-orientation) plays in impairing emotional decision behavior. The detrimental effects of impulsivity on the classic IGT have been reported elsewhere [4], [7]–[11]. Nevertheless, the small impact of impulsivity on the original task may be the reason why its damaging effects on IGT performance sometimes went undetected [5], [12], [13]. We found that low impulsive participants outperformed high impulsive participants in the last two blocks of trials, in particular. Neurophysiological evidence suggests that different brain systems are involved in tasks that require the individual to evaluate immediate and delayed rewards [28]. Specifically, the first stages of performance require an active reward system so that the subject can learn or identify the best long-term options, whereas the latter stages require an efficient cognitive self-control system to keep the allure of immediate rewards at bay. Consistent with this model, the high impulsive individuals' performance deterioration in the later IGT blocks suggests that they show a comparative deficit in their self-control systems' ability to maintain the choice of the best long-term options. Analyses of individual preferences for specific decks further supported the irrational decision making of high impulsive participants, who showed a “prominent deck B” effect in the IGT-v – that is, a preference for deck B selections [25]. In this regard, Takano and colleagues [10] found that implicitly impulsive participants – i.e., those whose impulsivity was assessed by a behavioral test – showed the same preference for deck B in a modified version of the IGT characterized by progressive changes in delayed punishments. Our results extend these findings and support the “prominent deck B” effect for self-rated impulsive individuals in another variant of the IGT.

The anticipated effect of sensation seeking was also confirmed, as this personality trait in fact exerted a negative influence in the IGT-v. This was probably due to the higher level of arousal associated with disadvantageous decks, which made them riskier and thus more attractive to high sensation seekers.

In contrast with our expectations, BAS facets appeared to exert little influence irrespective of IGT version, with the notable exception of Fun Seeking, which was found to negatively correlate with advantageous decision making in the IGT-v. Since this trait shares many features with both sensation seeking and impulsivity [21], the result is in line with the effects described above. As regards the other BAS facets, one possible reason for the null findings might be that the IGT, as well as the IGT-v, is less than ideally suited to highlight BAS involvement in decision making, due to the fact that it combines rewards, losses and risky outcomes (i.e., disadvantageous options were characterized not only by the greatest gains but also by the greatest losses). One possibility is that individuals with high Reward Sensitivity might have noted this co-occurrence and might have selected cards in order to preserve the long-term gains, regardless of variations in the reinforcement schedule. Indeed, unlike the Impulsivity dimension, which can be typified by the Non-Planning component, the core meaning of Reward Sensitivity is not directly associated with impaired planning skills; rather, it includes components such as reward responsiveness, persistence in the pursuit of rewards and fun seeking. Therefore, it is possible that the limited planning abilities, that drove more impulsive participants toward disadvantageous decisions, did not affect the choice behavior of individuals who scored high in Reward Sensitivity. Although this post-hoc hypothesis requires further investigation, recent findings are consistent with such a possibility [29], in that they show that gain amount manipulations selectively interact with BAS Reward Responsiveness in affective decision contexts in which reward amount, loss amount and loss probability are kept separate, through a full factorial design.

In line with our predictions, regression analyses have shown that high levels of BIS-Anxiety seem to improve performance on the IGT-v. It is likely that those who scored highly in this trait, by experiencing greater levels of anxiety in the highly punitive contingencies of disadvantageous decks, were more inclined to avoid them. This preliminary result, considered in conjunction with the effects in the opposite direction previously found for this trait in the original IGT (i.e., worse performance for more anxious individuals; e.g., [4], [30], [31]), may suggest that some variables in the decision context can be crucial in determining whether specific personality variables favor or impair decision making. Although further data are needed to validate this finding, such a pattern stresses the importance of the decision context variables (in this case, the magnitude of immediate punishments) in determining the extent to which specific personality traits can alter decision behaviors. In this respect, we think that a very fruitful approach in this field of research – especially given its clinical implications – could be the systematic investigation of the decision context parameters that are able to impact choice behaviors by interacting with specific personality traits (i.e., the magnitude and/or frequency of rewards and punishments). Indeed, under some circumstances, changes in such parameters might explain why individuals with specific personality constellations can switch from advantageous to disadvantageous decision making (or *vice versa*).

In conclusion, the current study shows that, by increasing the conflict between short- and long-term gains in the IGT paradigm, the effects exerted by personality on decision making are more likely to emerge. Further studies are needed to test the robustness of these preliminary findings and to estimate the external validity of the IGT-v in predicting other risk behaviors. If further data are found to be in line with our findings, we encourage the deployment of the IGT-v, in lieu of the original IGT, in future investigations of the impact of personality on healthy individuals' choice behavior. Indeed, the IGT-v entirely preserves the original task structure (i.e., it assessed affective decision making under conditions of ambiguity and risk), while at the same time it seems to be more sensitive than the original task version for disclosing personality influences on choice behavior.
